# Potential mental health-related harms associated with the universal screening of anxiety and depressive symptoms in Australian secondary schools

**DOI:** 10.1186/s13034-024-00734-y

**Published:** 2024-04-02

**Authors:** Taylor A. Braund, Simon T. E. Baker, Mirjana Subotic-Kerry, Gabriel Tillman, Nathan J. Evans, Andrew Mackinnon, Helen Christensen, Bridianne O’Dea

**Affiliations:** 1 Black Dog Institute, University of New South Wales, Randwick, NSW Australia; 2 Faculty of Medicine and Health, University of New South Wales, Sydney, NSW Australia; 3 Orygen, University of Melbourne, Melbourne, VIC Australia; 4 Institute of Health and Wellbeing, Federation University, Ballarat, VIC Australia; 5 School of Psychology, University of Queensland, St Lucia, QLD Australia

**Keywords:** Adolescent, Students, Harms, Screening, Anxiety, Depression, Schools, Intervention

## Abstract

**Background:**

Anxiety and depressive disorders typically emerge in adolescence and can be chronic and disabling if not identified and treated early. School-based universal mental health screening may identify young people in need of mental health support and facilitate access to treatment. However, few studies have assessed the potential harms of this approach. This paper examines some of the potential mental health-related harms associated with the universal screening of anxiety and depression administered in Australian secondary schools.

**Methods:**

A total of 1802 adolescent students from 22 secondary schools in New South Wales, Australia, were cluster randomised (at the school level) to receive either an intensive screening procedure (intervention) or a light touch screening procedure (control). Participants in the intensive screening condition received supervised self-report web-based screening questionnaires for anxiety, depression and suicidality with the follow-up care matched to their symptom severity. Participants in the light touch condition received unsupervised web-based screening for anxiety and depression only, followed by generalised advice on help-seeking. No other care was provided in this condition. Study outcomes included the increased risk of anxiety, depression, psychological distress, decreased risk of help-seeking, increased risk of mental health stigma, determined from measures assessed at baseline, 6 weeks post-baseline, and 12 weeks post-baseline. Differences between groups were analysed using mixed effect models.

**Results:**

Participants in the intensive screening group were not adversely affected when compared to the light touch screening condition across a range of potential harms. Rather, participants in the intensive screening group were found to have a decreased risk of inhibited help-seeking behaviour compared to the light touch screening condition.

**Conclusions:**

The intensive screening procedure did not appear to adversely impact adolescents’ mental health relative to the light touch procedure. Future studies should examine other school-based approaches that may be more effective and efficient than universal screening for reducing mental health burden among students.

*Trial registration* Australian and New Zealand Clinical Trials Registry (ACTRN12618001539224) https://anzctr.org.au/Trial/Registration/TrialReview.aspx?id=375821.

**Supplementary Information:**

The online version contains supplementary material available at 10.1186/s13034-024-00734-y.

## Introduction

Anxiety and depressive disorders can be chronic and disabling if not identified and treated early in the course of illness [21]. Universal mental health screening of adolescents has emerged as one potential method of the timely identification of young people in need of mental healthcare and support. By using validated questionnaires, screening can identify adolescents who may (a) have an increased risk of these illnesses or are experiencing subsyndromal symptoms so that preventive action can be taken, or (b) have undiagnosed, clinically significant levels of illness so that treatment can be administered. Using screening to triage adolescents into appropriate treatments may help to reduce symptoms and accelerate recovery, leading to improved future health outcomes [38]. There has been growing calls internationally for the broader uptake of mental health screening in settings where young people frequent, including health services and schools [16, 19, 48]. In 2016, the U.S. Preventive Services Task Force (USPSTF) recommended screening for major depressive disorder in adolescents aged 12 to 18 years [38] and recently [46] expanded this advice to include the screening for anxiety [45]. In Australia, the Federal government also recently invested over $10 million Australian dollars in a universal mental health and wellbeing screening tool for schools [9]. Conversely, the Royal Australian College of General Practitioners do not recommend routine screening for depression in adolescents in primary care [31]. While evidence-based practice calls for high quality evidence, a major challenge for policy makers in this domain is the limited informativeness of past trials for determining the potential harms of mental health screening in adolescents.

There is limited evidence from RCTs on the overall benefits of mental health screening programs in improving health outcomes, and an even greater gap in understanding the potential harms related to the screening process. The USPSTF recommendations were based on a review of 80 studies that evaluated the benefits or harms of screening for depression, anxiety, and suicide risk compared to ‘no screening’ or ‘usual care’. The evidence considered by the USPSTF primarily focused on the benefits and harms of exposing youth to anxiety and depression treatments and the accuracy of screening questionnaires, rather than the harms associated with other aspects of the screening processes such as the level of supervision, support, and intervention offered to participating youth [49]. Two systematic reviews Williams et al. [50] and Roseman et al. [36]) found no RCTs examining the effects of depression screening on outcomes for children and adolescents. This lack of evidence was further confirmed in a later review by Anderson et al. [2], who found only one RCT examining the utility of universal screening programs for youth mental health. Some recent RCTs have examined various screening approaches in schools, with mixed findings [14, 20, 27, 37]. Sekhar et al. [37] found that adolescents who underwent universal screening for depression at school were 5.9 times more likely to be detected, 3.3 times more likely to confirm the need for treatment, and 2.1 times more likely to start, when compared to targeted screening [14, 20, 27, 37]. This is consistent with Husky et al. [14], who also found that universal screening in schools led to a significantly greater proportion of youth being identified and referred to services than targeted screening. In a large, multi-site trial across six middle schools in the United States, Makover et al. [20] showed that universal screening was an effective method of detecting students who would benefit from a targeted depression intervention. In Australia, O'Dea et al. [27] found that a web-based screening platform with stepped-care interventions for depression and anxiety significantly improved adolescents’ likelihood of seeking help, but had no effect on their depressive symptoms. However, none of these studies explicitly examined the potential harms that may be associated with screening processes in adolescents.

When undertaken without adequate supervision and support, universal screening for depression and anxiety may induce distress through emotional activation and increased self-awareness of negative symptoms [35, 39]. Inaccurate screening results may fail to identify all those in need or may lead to unnecessary intervention and overtreatment, wasted time and resources, victimisation, stigma, isolation, shame, and negative stereotyping [2, 10, 35, 41]. Although universal mental health screening in schools is generally supported by parents and teachers [23, 28, 40], one in 10 parents have reported perceived harms due to lack of trained staff, and potential discomfort and stigmatisation of the student [40]. School counsellors have also reported specific concerns about parental agreement, students’ readiness for follow-up, and adequate resourcing of counsellors to care for all students in need [6, 26, 28]. These concerns have were validated in Sekhar and colleagues [37] large (*n* > 12 k) school-based screening trial for depression, which found that the primary reason for failure of treatment initiation among students was lack of parent or individual consent. Screening may provide schools with important data on the need for mental health programs and help to guide decision-makers in selecting and targeting such programs. For example, a US County Schools Mental Health Coalition uses the prevalence of positive cases identified through screening to determine whether universal (prevalence rate > 20%) or selective programs (prevalence rate < 15%) are required [32]. However, the level of potential harm associated with different screening procedures and components remains unclear. A growing number of research trials have shown that some school-based mental health programs do result in iatrogenic effects [4, 12, 22]. As school-based screening programs are resource intensive, in terms of time, personnel, infrastructure, equipment and training [13, 41], it is important for schools to carefully consider the suitability of these activities, to ensure that limited resources are being used effectively and efficiently to maximize benefits for students. It is vital for researchers and service designers to explicitly examine the potential harms of school-based screening with student mental health outcomes of paramount importance.

## Objectives of the current paper

**Fig. 1:**
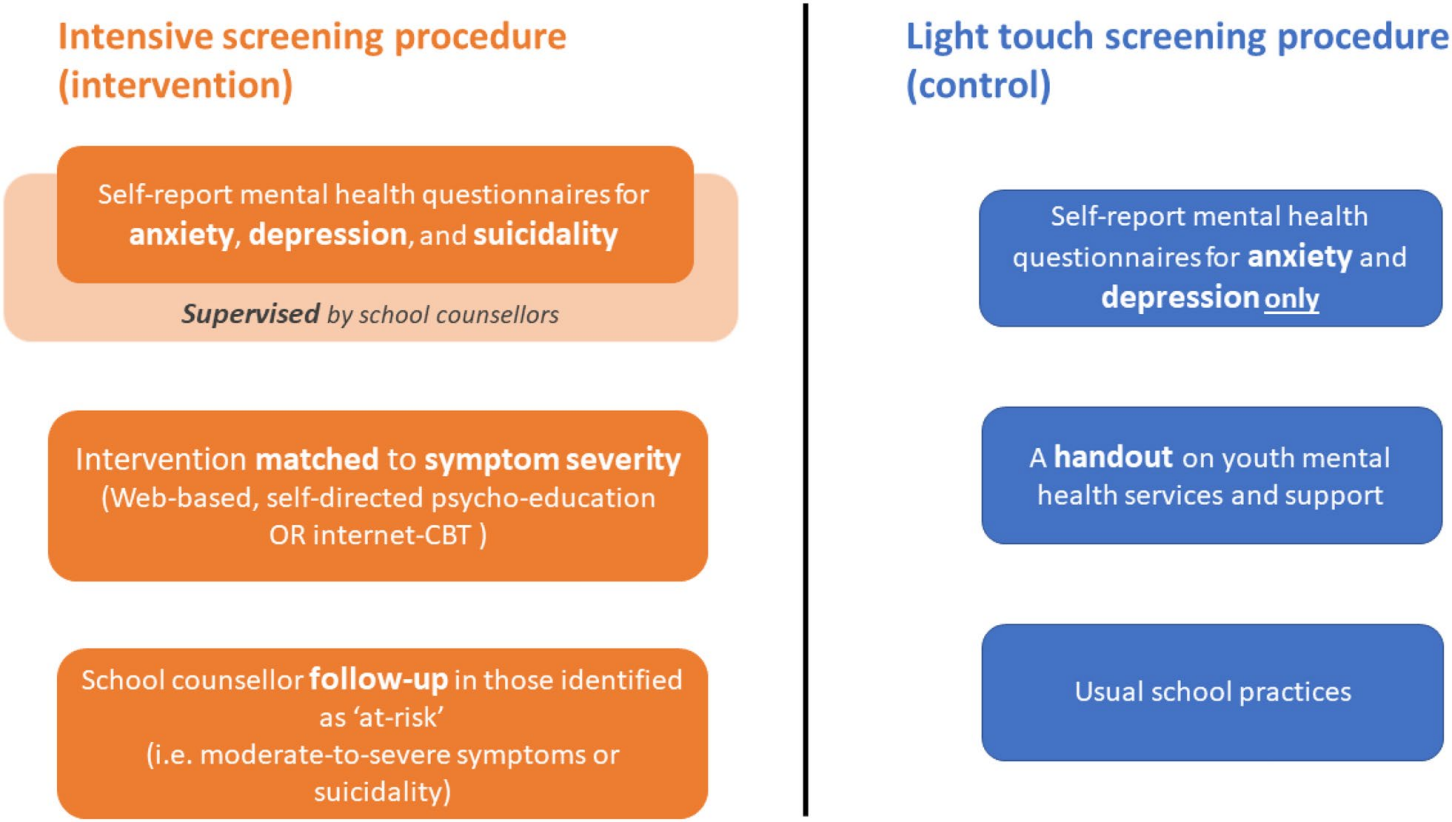
Brief overview of screening procedures examined in current study

This paper explores some of the potential mental health-related harms associated with the universal screening of anxiety and depression administered in Australian secondary schools as part of a cluster RCT that examined the effectiveness of a digital mental health service for improving adolescents’ help-seeking [25, 27, 29]. This paper presents a secondary data analysis of this recent RCT as its design presents a unique opportunity to compare whether a more intensive screening procedure for anxiety and depression is associated with greater risk of potential mental health-related harms when compared to a control condition that involved a light touch screening procedure (see Fig. 1). For example, schools have mandatory reporting procedures that are initiated when a person reports suicidality. As a result, the use of this additional measure may lead unintended harm to students by initiating this process.

In contrast to past work, this paper explicitly defines and measures student-level harms that are relevant to decision-makers wishing to compare school-based screening processes. In the current paper, the potential mental health-related harms examined were:

(i) The increased risk of anxiety and depressive symptoms and psychological distress;
(ii) The increased risk of deterioration in help-seeking and daily functioning;
(iii) The increased risk of mental health stigma.

Hypotheses were framed in terms of the more intensive screening procedure (i.e., intervention condition) being no worse than the light touch screening procedure (i.e., control condition) and inferences were primarily based on point estimates and confidence intervals rather than significance to determine the risk of harms [11, 44]. Based on previous findings [3, 35, 41], it was hypothesised that students who received the more intensive screening procedure would show no increase in risk of harm compared to students who received the ‘light touch’ procedure. These findings sought to provide the first evidence from an RCT on the potential mental health-related harms of universal screening and subsequent intervention for symptoms of anxiety and depression in the secondary school setting.

## Method

### Design

This paper is a secondary analysis of a two-arm 12 week cluster RCT. The trial was undertaken between February and December 2018 in secondary schools in New South Wales, Australia. Schools were the clusters and individual students were the participants [25, 27]. The full trial protocol [25] and primary outcomes [27] have been published elsewhere. All mental health-related harms examined in this paper pertain to the individual participant level.

### Participants

Secondary school adolescents (age range: 11–19 years) from grades (7–12) who attended participating schools were eligible to take part. A total of 1098 students from 12 schools were allocated to the control condition and 704 students from 10 schools were allocated to the intervention (CONSORT chart provided in Additional file 1: Fig. S1). Participation was voluntary and parents/guardians who did not wish for their child to take part were instructed to notify the school or research team prior to the baseline assessment. Students provided informed online consent during the baseline assessment in the presence of the research team and a school representative.

## Screening procedures

### Intensive screening procedure (Intervention condition)

As outlined in Fig. 1 and described in Additional file 1 the intensive screening procedure offered schools a comprehensive approach to universal screening, intervention and monitoring for anxiety and depression. Students in this condition completed the self-report screening measures for generalised anxiety and depressive symptoms using a web-based service platform called ‘Smooth Sailing’. This screening procedure involved an additional depressive symptom scale (Patient Health Questionnaire—9—Adolescent version, PHQ-9; [17], which included one item to assess students’ suicidality (i.e., thoughts of death and/or self-harm). Upon completion of the self-report screeners, the service then automatically recommended students complete activities that were matched to their symptom severity. The recommended activities were displayed to students on a personalised dashboard within the Smooth Sailing platform, which also provided generalised feedback on symptom severity. Using the thresholds of the GAD-7 and PHQ-9 screeners, students with ‘nil’ to ‘mild’ symptoms were recommended to complete a series of interactive, online, self-directed psycho-education modules that were hosted within the web-based platform. Students with ‘mild’ to ‘moderate’ symptoms were recommended to complete MoodGYM or BRAVE Online (two online, evidence-based, self-directed cognitive behavioural therapy programs for depression and anxiety in youth). Students with ‘moderately severe’ to ‘severe’ symptoms or who reported suicidality received an in-person follow-up with a school counsellor within 48 h. After 6 weeks, students repeated the mental health screening, and the level of intervention was then ‘stepped up’ in response to students’ deterioration. In this condition, all three screening sessions were conducted in the presence of a researcher, teacher, and school counsellor. The school counsellor was required to be onsite for 2 days after the screening sessions to ensure all students in need were attended to. School counsellors were given access to a purpose-built, secure online platform that enabled them to log in to identify and monitor the identified students. School counsellors were also provided with a list of local mental health service and supports that they could refer students to. The research team contacted all participating school counsellors after 48 h of the screening sessions to ensure all follow-ups had been conducted.

### Light touch screening procedure (Control condition)

Students in this condition completed the screening measures using an identical web-based platform to the screening procedure 1; however, the PHQ-9 scale (which included the measure on suicidality) was not administered. Upon completion of the screening measures, students in this condition were not given access to any of the additional processes or components (i.e., symptom feedback, psycho-education modules, online cognitive behavioural therapy program, follow-ups with the school counsellor). Instead, all students were displayed information on a range of different youth mental health services and support (including in-person, web-based and telephone services). This information was also provided to students as a one-page printed handout. In this arm, the screening procedure was conducted in the presence of a researcher and schoolteacher. The school counsellor was required to be onsite only on the day of screening but did not attend the screening sessions. No additional contact was made with the schools after each screening session. No limitations were placed on these students’ mental healthcare activities, practices, or help-seeking during the study period.

## Measures of mental health-related harms

### Depressive symptoms

Depressive symptoms were measured using the Centre for Epidemiologic Studies Depression Scale—Child version (CES-DC) [34]. This 20-item self-report scale was administered at baseline and 12 weeks post-baseline. Item scores were summed, with higher scores indicative of greater depressive symptoms. For the sample included in this paper, participants’ total scores ranged from 0 to 60 and Cronbach’s alpha was 0.93. For this paper, total scores ≥ 16 were classified as clinically meaningful cases and ‘new’ cases were participants who emerged as a case only at week 12.

### Anxiety symptoms

Anxiety symptoms were measured using the Generalised Anxiety Disorder Questionnaire (GAD-7) [42]. This 7-item self-report scale was administered at baseline and 12 weeks post-baseline. Item scores were summed, with higher scores indicative of greater anxiety with the following score descriptors: ‘nil to mild’ (0–9), ‘moderate’ (10–14), or ‘moderately severe to severe’ (≥ 15). For the sample included in this paper, participants’ total scores ranged from 0 to 21 and the Cronbach’s alpha was 0.89. For this paper, total scores ≥ 10 were classified as clinically meaningful cases and ‘new’ cases were participants who emerged as a case only at week 12.

### Psychological distress

Psychological distress was measured using the Distress Questionnaire-5 (DQ5) [5]. This 5-item self-report scale was administered at baseline and 12 weeks post-baseline. Item scores were summed, with higher scores indicative of greater psychological distress. For the sample included in this paper, total scores ranged from 5 to 25 and the Cronbach’s alpha was 0.88. For this paper, total scores ≥ 14 were classified as clinically meaningful cases [5] and ‘new’ cases were participants who emerged as a case only at week 12.

### Help-seeking intentions and behaviour

The General Help-Seeking Questionnaire (GHSQ) [51] was used to measure participants’ intentions to seek help for general mental health problems. This was administered at baseline and 12 weeks post-baseline. Using a 5-point Likert scale, participants were asked to rate how likely they were to seek help from 13 sources when having a tough time with their mental health. Item scores were summed, with higher scores indicative of greater intentions to seek help. For the sample included in this paper, the total scores ranged from 13 to 65 and the Cronbach’s alpha was 0.87. The Actual Help-Seeking Questionnaire (AHSQ) [33] was used to measure help-seeking behaviour for mental health among participants. Participants were asked whether they had turned to the same list of sources outlined in the GHSQ for help with any mental health issue in the past three months (answered yes or no). For this paper, the 12-week assessment was utilised and participants were classified as an ‘inhibited help-seeker’ based on whether they self-identified as needing support for their mental health but did not seek help from anyone (i.e. those who answered yes to the final item of AHSQ “I needed support but I did not seek help from anyone”).

### Mental health stigma

This was measured using the Mental Health Literacy Scale (MHLS) [24]. This 13-item composite scale measured students’ confidence in seeking help (4 items) and their level of stigmatising attitudes towards mental illness (9 items) at baseline and 12 weeks post-baseline. Items were rated on a 5-point Likert scale. Item scores were summed, with higher total scores indicative of greater confidence in help-seeking and lower levels of stigma. The MHLS has demonstrated good internal and test–retest reliability and has been used in school-based mental health research [47]. For the sample included in this paper, participants’ scores ranged from 13 to 65 and the Cronbach’s alpha was 0.71.

### Daily functioning

To determine the functional impairment caused by their mental health problems, participants were asked to rate “how difficult have your mental health problems made it for you to do your schoolwork, take care of things at home, or get along with your mates and family?” using a 4-point Likert scale with answers ranging from “not difficult at all” to “extremely difficult”. This question is a supplementary item within the adolescent version of the PHQ-9 scale [17]. In this sample, participants scores ranged from 0 to 3. For this paper, the 12-week assessment was utilised and participants were classified as ‘Not impaired’ (i.e., those who responded, “not difficult at all” or “somewhat difficult”) or ‘Impaired’ (i.e., those who responded, “very difficult” or “extremely difficult”).

## Reliable change scores

As the GHSQ and MHLS do not have established cut-points, reliable change (RC) scores were calculated to assess meaningful individual-level change. Individual difference scores were calculated by subtracting the total score at baseline (${{\text{X}}}_{1}$) from the total score at 12 weeks post-baseline (${{\text{X}}}_{2}$). These difference scores were then used to calculate RC scores (for formulas used to calculate RC scores, see Additional file 1: Text S2). ${\text{RC}}\le -1.96$ and ${\text{RC}}\ge 1.96$ indicate a statistically significant difference from the average difference score and therefore reflect a “reliable change.”

## Harms analysis

Case classification, reliable change, and other classifications were then used to calculate several measures such as risk ratios and risk differences. Equations for each of these measures are presented in Additional file 1: Table S1 and were computed for each outcome by comparing the occurrence of that outcome among students in the screening condition (i.e., Experimental Event Rate [EER]) to its occurrence among students in the control condition (i.e., Control Event Rate [CER]).

## Statistical analyses

All analyses were conducted using Stata 17.0 [43]. Group differences between the conditions at baseline were examined using mixed linear or logit models. Baseline characteristics were included as the dependent variables, condition was included as a fixed effect, and school was included as a cluster random effect. Poisson generalised linear models with robust standard errors were used to estimate the relative risk of harmful outcomes between the conditions, which have shown to outperform log binomial models when calculating risk ratios [8, 52]. Outcomes (i.e., case classifications, reliable change scores, and other classifications) were included as the dependent variables, condition was included as a fixed effect, and school was included as a cluster random effect. Population average estimates were used instead of cluster specific estimates to control for the effects over clusters. Intracluster correlation coefficients (ICC) were calculated to examine the impact of clustering. The ∞ component of the confidence intervals for relative risk of harmful outcomes indicates instances where the statistical test is non-significant. Specifically, these instances occur when transforming both positive and negative values (and hence spans zero), resulting in confidence intervals that encompass two disjointed regions (e.g., NNTB to ∞, NNTH to ∞). As a result, confidence intervals were expressed as number needed to treat for harm (NNTH) to ∞ to number need to treat for benefit (NNTB) for continuity, in line with Altman [1].

## Results

### Participants

Participant characteristics are presented in Table 1. No significant differences were found between the intensive screening group and light touch group on baseline sample characteristics. Case classification frequencies are presented in Table 2 and reliable change and other classification frequencies are presented in Table 3.

**Table 1:** Baseline sample characteristics for the sample (*N* = 1802)

	Total sample (*N* = 1802)		Light touch screening procedure (control) (*n* = 1098)		Intensive screening procedure (intervention) (*n* = 704)		*p*
	*M*	*SD*	*M*	*SD*	*M*	*SD*	
Age	14.30	0.86	14.12	0.89	14.59	0.75	0.088
Depressive symptoms (CES-D)	16.43	12.28	17.40	12.53	14.91	11.74	0.333
Generalised anxiety (GAD-7)	5.97	5.20	6.51	5.23	5.13	5.02	0.071
Psychological distress (DQ5)	11.04	4.94	11.44	4.94	10.41	4.88	0.201
Help-seeking intentions (GHSQ)	34.38	10.29	34.43	10.16	34.31	10.51	0.925
Mental health stigma (MHLS)	34.47	6.85	34.69	6.91	34.13	6.74	0.963

	*n*	%	*n*	%	*n*	%	
Female	930	51.6	571	52.0	359	51.0	0.626
Needed support for mental health but did not seek help from anyone in the past 3 months (AHSQ)	325	18.1	215	19.6	110	15.7	0.372
Impaired daily functioning due to mental health	277	15.4%	186	16.9	91	12.9	0.508

**Table 2:** Case Classifications at baseline, 6 weeks post-baseline, and 12 weeks post-baseline

	Light touch screening procedure (control)															Intensive screening procedure (intervention)														
	Baseline					6 weeks					12 weeks					Baseline					6 weeks					12 weeks				
	Non-case		Case		Total	Non-case		Case		Total	Non-case		Case		Total	Non-case		Case		Total	Non-case		Case		Total	Non-case		Case		Total
	*n*	%	*n*	%	*n*	*n*	%	*n*	%	*n*	*n*	%	*n*	%	*n*	*n*	%	*n*	%	*n*	*n*	%	*n*	%	*n*	*n*	%	*n*	%	*n*
Depression (CES-DC)	601	54.7	497	45.3	1098	543	58.1	391	41.9	934	515	58.7	362	41.3	877	444	63.2	258	36.8	702	291	65.7	152	34.3	443	267	67.1	131	32.9	398
New case^a^	–	–	–	–	–	–	–	–	–	–	–	–	34	4.3	793	–	–	–	–	–	–	–	–	–	–	–	–	13	4.1	316
Anxiety (GAD-7)	803	73.1	295	26.9	1098	739	79.0	197	21.0	936	679	77.3	199	22.7	878	576	81.8	128	18.2	704	387	86.2	62	13.8	449	347	86.8	53	13.3	400
New case^a^	–	–	–	–	–	–	–	–	–	–	–	–	37	4.6	796	–	–	–	–	–	–	–	–	–	–	–	–	13	4.1	320
Psychological distress (DQ-5)	744	67.8	354	32.2	1098	671	71.8	264	28.2	935	622	70.8	256	29.2	878	526	74.9	176	25.1	702	353	79.5	91	20.5	444	319	80.2	79	19.8	398
New case^a^	–	–	–	–	–	–	–	–	–	–	–	–	40	5.0	795	–	–	–	–	–	–	–	–	–	–	–	–-	10	3.2	317

*Note:* Totals vary due to missing data and loss to follow-up

*CES-DC* Centre for Epidemiologic Studies Depression Scale, *GAD-7* Generalised Anxiety Disorder Questionnaire-7, *DQ-5* Distress Questionnaire-5

^a^ New case refers to students who were not classified as a case at either of the previous time points (i.e., baseline or 6 weeks post-baseline)

**Table 3:** Reliable change and other classifications

	Light touch screening procedure (control)							Intensive screening procedure (intervention)						
	Reliable deterioration		No reliable change		Reliable improvement		Total	Reliable deterioration		No reliable change		Reliable improvement		Total
	*n*	%	*n*	%	*n*	%	*n*	*n*	%	*n*	%	*n*	%	*n*
Help-seeking intentions (GHSQ)	34	3.9	820	93.5	23	2.6	877	8	2.0	374	94.2	15	3.8	397
Mental health stigma (MHLS)	26	3.0	818	94.1	25	2.9	869	7	1.8	379	96.4	7	1.8	393

	Did not seek help				Sought help		Total	Did not seek help				Sought help		Total
	*n*	%	–	–	*n*	%	*n*	*n*	%	–	–	*n*	%	*n*
Inhibited help-seeking (AHSQ)	141	16.1	–	–	735	83.9	876	32	8.1	–	–	363	91.9	395

	Impaired				Not impaired		Total	Impaired				Not Impaired		Total
	*n*	%	–	–	*n*	%	*N*	*n*	%	–	–	*n*	%	*n*
Daily functioning	121	13.8	–	–	757	86.2	878	39	9.7	–	–	362	90.3	401

*Note:* Totals vary due to missing data and loss to follow-up and comprise students with data available at both the baseline time point and the 12-week post-baseline time point

*GHSQ* General Help-Seeking Questionnaire, *MHLS* Mental Health Literacy Scale, *AHSQ* Actual Help-Seeking Questionnaire

### Risk measures

Event rates and risk indices for each outcome classification are presented in Table 4. Intensive screening was associated with a decreased risk of experiencing inhibited help-seeking behaviour (Relative Risk Reduction = 50%, 95% CI [27%, 74%]; Absolute Risk Reduction = 9%, 95% CI [3%, 15%]) with the NNTB indicating that 11 (95% CI [3, 19]) students needed to be screened to prevent 1 case of inhibited help-seeking behaviour. No other confidence intervals crossed the 0 threshold for risk ratios or ∞ for NNTB.

**Table 4:** Risk measures for each outcome classification

	${n/n}_{{\text{C}}}$	${n/n}_{{\text{E}}}$	CER	EER	RR	95% CI	*P*	ICC	*P*	RRR	95% CI	RRI	95% CI	*P*	ARR	95% CI	ARI	95% CI	*P*	NNTB	95% CI
Case classification																					
Depression (CES-DC)	362/877	131/398	0.413	0.329	0.80	[0.58, 1.10]	0.167	0.064	< 0.001	0.20	[− 0.05, 0.46]	− 0.20	[0.05, − 0.46]	0.121	0.08	[− 0.06, 0.15]	− 0.08	[0.06, − 0.15]	0.170	11	[NNTH 4 to ∞ to NNTB 27]
New case^a^	34/834	13/378	0.039	0.033	0.81	[0.43, 1.52]	0.511	0.034	0.391	0.19	[− 0.32, 0.70]	− 0.19	[0.32, − 0.70]	0.464	0.01	[− 0.02, 0.03]	− 0.01	[0.02, − 0.03]	0514	130	[NNTH 261 to ∞ to NNTB 521]
Anxiety (GAD-7)	199/878	53/400	0.227	0.133	0.61	[0.35, 1.08]	0.092	0.124	< .001	0.39	[0.03, 0.74]	− 0.39	[− 0.03, − 0.74]	0.030	0.10	[− 0.01, 0.21]	− 0.10	[0.01, − 0.21]	0.078	10	[NNTH 1 to ∞ to NNTB 21]
New case^a^	37/823	13/373	0.043	0.034	0.78	[0.53, 1.15]	0.210	0.000	1	0.22	[− 0.08, 0.52]	− 0.22	[0.08, − 0.52]	0.156	0.01	[− 0.01, 0.02]	− 0.01	[0.01, − 0.02]	0.213	107	[NNTH 61 to ∞ to NNTB 275]
Psychological distress (DQ-5)	256/878	79/398	0.292	0.198	0.66	[0.42, 1.06]	0.084	0.112	< .001	0.34	[0.03, 0.65]	− 0.34	[− 0.03, − 0.65]	.032	0.11	[− 0.01, 0.23]	− 0.10	[0.01, − 0.23]	0.071	9	[NNTH 1 to ∞ to NNTB 18]
New case^a^	40/817	10/370	0.047	0.026	0.50	[0.19, 1.32]	0.160	0.045	0.278	0.50	[0.02, 0.99]	− 0.50	[− 0.02, − 0.99]	0.042	0.02	[− 0.01, 0.05]	− 0.02	[0.01, − 0.05]	0.110	41	[NNTH 9 to ∞ to NNTB 90]
Reliable change classification																					
Help-seeking intentions (GHSQ)	34/877	8/397	0.039	0.020	0.50	[0.18, 1.42]	0.193	0.094	0.009	0.50	[− 0.02, 1.02]	− 0.50	[0.02, − 1.02]	0.060	0.02	[− 0.01, 0.05]	− 0.02	[0.01, − 0.05]	0.178	50	[NNTH 23 to ∞ to NNTB 122]
Mental health stigma (MHLS)	26/869	7/393	0.030	0.018	0.61	[0.24, 1.53]	0.286	0.023	0.679	0.39	[− 0.17, 0.95]	− 0.39	[0.17, − 0.95]	0.170	0.01	[− 0.01, 0.03]	− 0.01	[0.01, − 0.03]	0.248	85	[NNTH 60 to ∞ to NNTB 231]
Other classification																					
Inhibited help-seeker (AHSQ)	141/876	32/395	0.161	0.081	0.50	[0.31, 0.80]	0.004	0.068	< .001	0.50	[0.27, 0.74]	− 0.50	[− 0.27, − 0.74]	< 0.001	0.09	[0.03, 0.15]	− 0.09	[− 0.03, − 0.15]	< 0.005	11	[3, 19]
Impaired daily functioning	121/878	39/401	0.138	0.097	0.73	[0.43, 1.23]	0.223	0.088	< .001	0.27	[− 0.11, 0.65]	− 0.27	[0.11, − 0.65]	0.160	0.04	[− 0.03, 0.11]	− 0.04	[0.03, − 0.11]	0.244	24	[NNTH 16 to ∞ to NNTB 65]

*Note:* Denominators (${n}_{{\text{C}}}$ and ${n}_{{\text{E}}}$) vary due to missing data and loss to follow-up. Models account for cluster (school) in log Poisson generalised linear mixed models.

${n}_{{\text{C}}}$ Total number of students in the control condition with data available for the specified measure, ${n}_{{\text{E}}}$ Total number of students in the intervention (experimental) condition with data available for the specified measure, *CER* Control Event Rate, *EER* Experimental Event Rate, *RR* Relative Risk, *RRR* Relative Risk Reduction, *RRI* Relative Risk Increase, *ARR* Absolute Risk Reduction, *ARI* Absolute Risk Increase, *NNT* Number Needed to Treat.

^a^ New case refers to students who were not classified as a case at either of the previous time points (i.e., baseline or 6 weeks post-baseline). ICC values was calculated using non-robust standard errors due to limitations in ICC calculations with robust standard errors

## Discussion

This paper examined some of the potential mental health-related harms associated with two types of universal school-based screening procedures for anxiety and depression in Australian adolescents using data from a cluster RCT. Given the lack of research in this field, this investigation aimed to provide additional insights to help policy-makers and researchers determine best-practice for school-based mental health screening in Australia and worldwide. Notably, there were a high number of cases of anxiety, depression, and psychological distress in the whole sample at baseline, which offers some support to the USPSTF’s recommendation of the need for universal screening for depression and anxiety in adolescents. In the current study, students who received the more intensive screening procedure were not adversely affected when compared to those who received the light touch procedure on a range of potential mental health-related harms, including the increased risk of clinically significant symptoms of anxiety, depression, and psychological distress or the deterioration in help-seeking intentions or mental health stigma. This is an important finding, given the more intensive screening procedure involved the assessment of suicidality, the provision of symptom feedback, and mandated follow-ups with school counsellors, which may have introduced the potential for greater risk. Overall, these findings suggest that the more intensive procedure did not appear to harm students on the measures collected.

In this paper, we investigated two types of screening procedures that differed in the intensity of care and intervention provided to students as well as the resources required by the schools. We found that the more intensive screening procedure resulted in a significantly lower risk of inhibited help-seeking behaviour in students. This is consistent with the primary outcomes of the effectiveness trial [27] and is an important finding given that inhibited help-seeking behaviour (i.e., having a self-identified need for mental health support but not actively seeking help) prolongs mental distress and delays treatment gains. This finding is consistent with Sekhar et al. [37] and indicates that direct links to accessible care are required for universal screening programs to shift help-seeking outcomes in adolescents. As the positive impacts of screening on health outcomes are mitigated by individuals’ willingness to engage in the treatment provided [15], careful consideration must also be given to the interventions recommended by adolescent screening programs. In this study, adolescents’ preferences for digital therapies (e.g., increased privacy, accessibility, autonomy) may have moderated the positive effects of the intensive screening procedure on help-seeking [30]. Different levels of harm may be found when the integrated treatments do not align with adolescents’ expectations, needs and or accessibility requirements. Furthermore, the interventions provided by screening programs may also have indirect effects on adolescent mental health. In this study, the intense screening procedure may have increased students’ awareness to and openness to other school-based supports (e.g. the school counsellor) but inverse effects may be found with different screening procedures. Future trials would also benefit from extrapolating the direct and indirect effects of screening processes on adolescent health outcomes and using qualitative inquiry to capture other types of experiences that are not easily measured through questionnaires.

While students who received the more intensive screening procedure were not adversely affected on any of the mental health harms examined when compared to the light touch procedure, the more intensive procedure required significantly more resources from both the students and schools, even with the integration of self-directed digital interventions. For example, students needed to allocate more time to this approach and required ongoing access to a computer and the Internet for completion of the digital content. School counsellors were also required to allocate significantly more time to completing training on the screening procedure and portal, supervising the screening practices, and following up all students who required it. As time, resources and costs impede the uptake of school-based mental health identification programs [13, 41], the intensive procedure may therefore not be feasible for all schools, despite low levels of harm. The few studies (none from Australia) that have examined the cost-effectiveness of school-based mental health screening approaches have had mixed findings [2, 7, 18]. Future studies should therefore aim to quantify the direct costs of universal screening programs for mental health in the Australian context, in comparison with other initiatives, so that schools are better placed to evaluate program affordability, sustainability and cost versus benefits.

## Limitations

This study was a secondary analysis of a cluster RCT and was not specifically designed to test for harms. As such, the findings are exploratory in nature. Further, as screening is likely to generate greatest benefit when delivered ‘en masse’, different levels of harms may emerge when larger samples from more schools are exposed to universal screening. Notably, data on other important potential harms (e.g., impact of false-positive and false-negative results, lack of treatment uptake and non-adherence, negative treatment effects) were not available for this study. While a single item was used to assess daily functioning for brevity and minimal participant burden, a more comprehensive assessment would benefit from the possibility of deeper insights into how their symptoms have impacted their daily life and relationships. The relatively short study period also limited the examination of long-term harms. There may be flow on effects of the negative experiences of universal screening on students, such as disengagement in care and reluctance to participate in other school-based mental health programs. In addition, as this study relied on voluntary participation, different levels of harms may emerge when screening is compulsory. Lastly, as suggested by Foulkes and Stringaris [12], there may be subgroups of adolescents who will experience harms from screening-related interventions, which may be masked when findings are averaged. There may also be harms associated with the different components of universal screening programs, such as the types of treatment young people are referred to, that are better captured through qualitative research.

## Conclusion

Our findings suggest that the intensive screening procedure examined by this paper did not appear to increase mental health-related harms for adolescents when compared to the light touch procedure. The intensive screening procedure may offer a beneficial approach for improving aspects of help-seeking behaviour. These results may alleviate some of the concerns held by schools, parents, and students on the benefits of screening and may facilitate greater participation in these procedures. However, given the high resource burden of the intensive screening, future studies are needed to determine whether this approach is superior to other school-based interventions for improving adolescents’ mental health. Moreover, future studies on school-based mental health screening should routinely define, assess, and report on potential harms over extended periods to comprehensively evaluate the impact and net benefit for students.

### Supplementary Information

**Additional file1: Figure S1.** Consort Chart. **Table S1.** Risk Measures and Equations. **Text S1.** Description of Smooth Sailing. **Text S2.** RC Score Calculation.

## Data Availability

The data collected and analysed in the current trial is not currently available to researchers outside of the approved team due to constraints placed on the project by the various ethics bodies. Additional related project documents are currently available from the web-based Australian and New Zealand Clinical Trials Registry.
