# Supplementary material for: Potential mental health-related harms associated with the universal screening of anxiety and depressive symptoms in Australian secondary schools
Source: Child Adolesc Psychiatry Ment Health. 2024 Apr 2;18:46. doi: 10.1186/s13034-024-00734-y (PMC10985850; doi:10.1186/s13034-024-00734-y)
Supplement: Supplementary file 1 — Additional file1: Figure S1. Consort Chart. Table S1. Risk Measures and Equations. Text S1. Description of Smooth Sailing. Text S2. RC Score Calculation. [file 13034_2024_734_MOESM1_ESM.docx]

**Additional file Material**

Additional file 1: Figure 1.

Consort Chart.


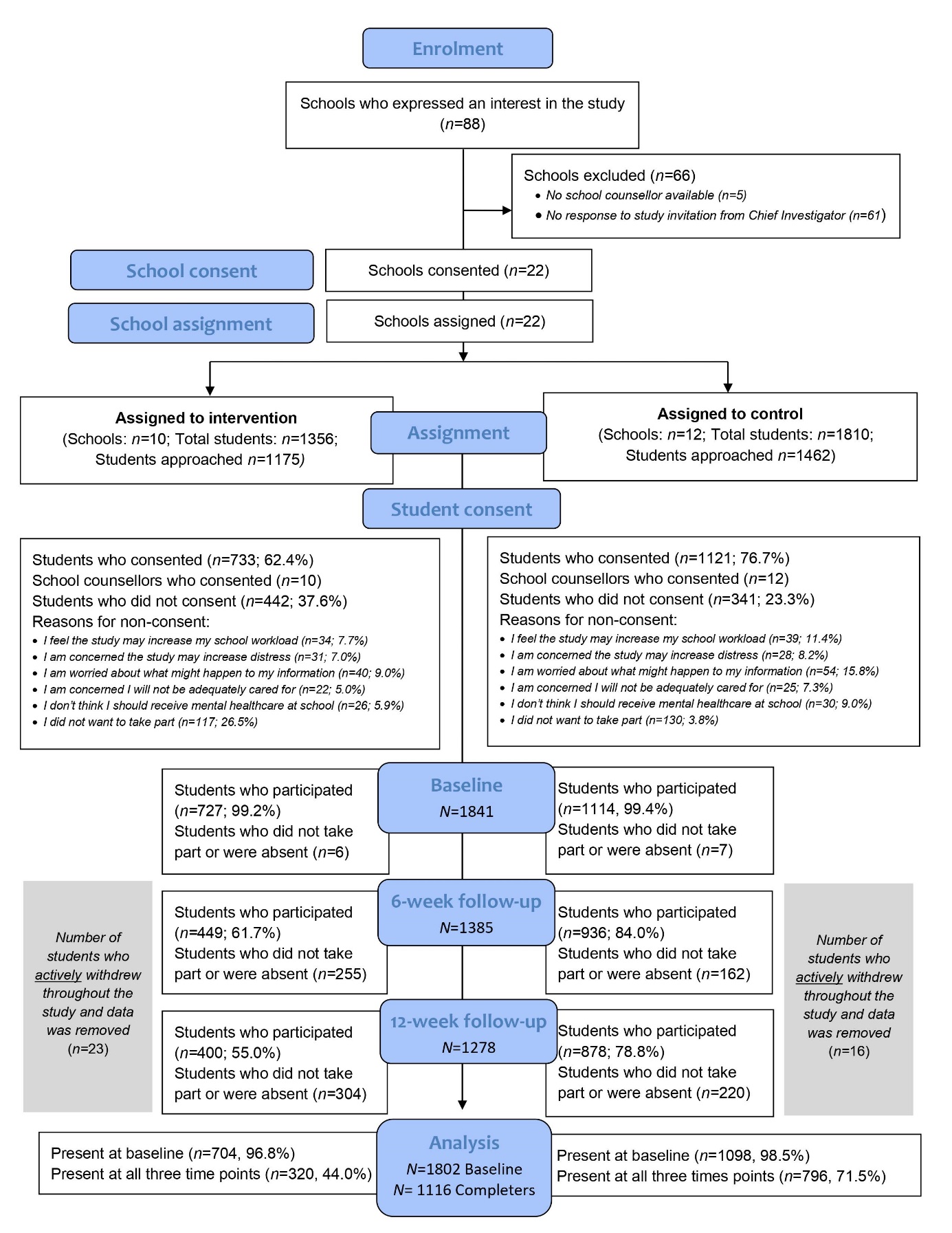


Additional file 1: Table 1.

Risk Measures and Equations.

| Measure | Equation |
| --- | --- |
| Control Event Rate (CER) | $CER=\frac{\mathrm{Events}_{C}}{n_{C}}$ |
| Experimental Event Rate (EER) | $EER=\frac{\mathrm{Events}_{E}}{n_{E}}$ |
| Risk Ratio (RR) | $RR=\frac{\mathrm{EER}}{\mathrm{CER}}$ |
| Relative Risk Reduction (RRR) | $RRR=\frac{CER-EER}{\mathrm{CER}}$ |
| Relative Risk Increase (RRI) | $RRR=\frac{EER-CER}{\mathrm{CER}}$ |
| Absolute Risk Reduction (ARR) | $ARR=CER-EER$ |
| Absolute Risk Increase (ARI) | $ARI=EER-CER$ |
| Number Needed to Treat for an Additional Beneficial Outcome (NNTB) | $NNTB=\frac{1}{\mathrm{ARR}}$ |
| Number Needed to Treat for an Additional Harmful Outcome (NNTH) | $NNTH=\frac{1}{\mathrm{ARI}}$ |

Note: The Relative Risk Reduction and Relative Risk Increase re-express the Risk Ratio (also known as the Relative Risk) as a percentage reduction and percentage increase, respectively. The Absolute Risk Reduction and Absolute Risk Increase are often collectively referred to as the Risk Difference and they re-express the absolute difference between the Control Event Rate and the Experimental Event Rate as a percentage reduction and percentage increase, respectively. Risk reduction measures, including Number Needed to Treat for an Additional Beneficial Outcome, are the additive inverse of their corresponding risk increase measures, including Number Needed to Treat for an Additional Harmful Outcome. Although the Number Needed to Screen [58] is sometimes used to quantify the utility of screening programs, it is based on the same formula as the Number Needed to Treat for an Additional Beneficial Outcome, which makes it redundant. Abbreviations: $\mathrm{Events}_{C}$, Number of students in the control condition who experienced the event of interest; $n_{C}$, Total number of students in the control condition with data available for the outcome measure of interest; $\mathrm{Events}_{E}$, Number of students in the intervention (experimental) condition who experienced the event of interest; $n_{E}$ Total number of students in the intervention (experimental) condition with data available for the outcome measure of interest.

Additional file 1: Text 1

Description of Smooth Sailing.

**Smooth Sailing**

Developed by researchers at the Black Dog Institute, mental health professionals, young people and school staff, Smooth Sailing is a web-based mental health service for secondary schools. The service offers a comprehensive approach to the identification of students in need of care for anxiety and depression, direct links to evidence-based interventions, ongoing monitoring and stepping up of care in response to symptom deterioration. The mental health screening within Smooth Sailing consisted of the Generalised Anxiety Disorder Scale (GAD-7) and the Patient Health Questionnaire (PHQ-9) which determined students’ symptom levels. Using the total scores of the GAD-7 and PHQ-9, the service allocated students to a step of care and provided students with a personalised dashboard that included generalised symptom feedback and an overview of the recommended activities. The self-directed, web-based psychoeducation consisted of five 10-minute self-directed modules on general mental health, anxiety, depression, and help-seeking which were complimented by animations, illustrations, and hyperlinks to credible youth mental health services and websites. An additional module provided referral to two external, publicly accessible, free, evidence-based Internet Cognitive Behaviour Therapy (iCBT) programs for youth provided by Australian universities: MoodGym and The BRAVE Program. These iCBT programs were provided external to the Smooth Sailing service and thus, the research team did not have access to the data collected by these programs. All module content was created specifically for the Smooth Sailing service and was reviewed by youth and health professionals in the co-design process. The content was also edited by a copywriter to ensure appropriate readability for young adolescents. The Smooth Sailing service also included a notification system to link students to their school counsellors. Students with ‘moderately severe’ to ‘severe’ symptoms and/or experiencing thoughts that that they would be better off dead or of hurting themselves (i.e., score > 0 on item 9 of the PHQ-9) triggered an immediate follow-up notification. School counsellors accessed and tracked these notifications using the purpose-built secure web-portal. School counsellors were provided with information guides to support the interpretation of students’ mental health scores and were instructed to use their school protocols when attending to students, initiating external referrals or parental contact when necessary. School counsellors were provided with a list of local mental health services to assist this process. To ensure student confidentiality, the researchers were not provided with specific details on the actions taken or care provided by the school counsellors. As part of the Smooth Sailing service, all students were invited to complete fortnightly mood check-ins sent via short message services (SMS) or email. This check-in consisted of the GAD-2 and PHQ-2 symptom scales. A total of four check-ins were sent during the study period. Students received generalised feedback and were prompted to engage in their recommended activities. All students were also sent fortnightly reminders to complete their activities. The mental health screening was repeated at 6- and 12-weeks post-baseline, and any student who had not responded to their allocated care (i.e., symptoms remained elevated or had worsened) were stepped up to the next level of care. Due to the novelty of the service model and the short study period, no students were stepped down.

Additional file 2: Text 2

RC Score Calculation.

RC scores were calculated as follows [59].

$$RC=\frac{X_{2}-X_{1}}{\sigma_{\mathrm{diff}}}$$

Where $\sigma_{\mathrm{diff}}$ is the standard error of measurement of the difference, calculated according to the method proposed by Christensen and Mendoza [59] (see Perdices [60], for alternatives), as follows.

$$\sigma_{\mathrm{diff}}=\sqrt{\sigma_{X_{1}}^{2}+\sigma_{X_{2}}^{2}-{2\sigma}_{X_{1}}\sigma_{X_{2}}r_{X_{1}X_{2}}}$$

Where $\sigma_{X_{1}}$ is the standard deviation of observed scores at the baseline time point, $\sigma_{X_{2}}$ is the standard deviation of observed scores at the 12-weeks post-baseline time point, and $r_{X_{1}X_{2}}$ is the Pearson correlation coefficient between baseline scores and 12-weeks post-baseline scores.
